# Fibroblast Growth Factor 11 Inhibits Hepatitis B Virus Gene Expression Through FXRα Suppression

**DOI:** 10.1007/s12275-023-00065-1

**Published:** 2023-08-30

**Authors:** Mi So Seong, Jeong Ah Jang, Ye Rim Jeong, Ye Bin Kim, Yi Yi Kyaw, Hee Jeong Kong, Jung-Hyun Lee, JaeHun Cheong

**Affiliations:** 1 Department of Molecular Biology, Pusan National University, Busan, 46241 Republic of Korea; 2 Advanced Molecular Research Centre, Department of Medical Research, Republic of Union of Myanmar, Yangon, 11191 Myanmar; 3 Biotechnology Research Division, National Institute of Fisheries Science, Busan, 46083 Republic of Korea; 4 Marine Biotechnology Research Center, Korea Institute of Ocean Science and Technology, Busan, 49111 Republic of Korea

**Keywords:** FGF11, Hepatitis B virus, HBx protein, FXR, Antiviral effect

## Abstract

Fibroblast growth factor 11 (FGF11) is a member of the intracellular FGF family, which shows different signal transmission compared with other FGF superfamily members. The molecular function of FGF11 is not clearly understood. In this study, we identified the inhibitory effect of FGF11 on hepatitis B virus (HBV) gene expression through transcriptional suppression. FGF11 decreased the mRNA and protein expression of HBV genes in liver cells. While the nuclear receptor FXRα1 increased HBV promoter transactivation, FGF11 decreased the FXRα-mediated gene induction of the HBV promoter by the FXRα agonist. Reduced endogenous levels of FXRα by siRNA and the dominant negative mutant protein (aa 1–187 without ligand binding domain) of FXRα expression indicated that HBV gene suppression by FGF11 is dependent on FXRα inhibition. In addition, FGF11 interacts with FXRα protein and reduces FXRα protein stability. These results indicate that FGF11 inhibits HBV replicative expression through the liver cell-specific transcription factor, FXRα, and suppresses HBV promoter activity. Our findings may contribute to the establishment of better regimens for the treatment of chronic HBV infections by including FGF11 to alter the bile acid mediated FXR pathway.

## Introduction

Hepatitis B virus (HBV) infection is a serious public health problem, affecting approximately 350 million people with chronic HBV infection worldwide. HBV infection is associated with a high risk of developing serious liver diseases including acute and chronic hepatitis, cirrhosis, and hepatocellular carcinoma (Ganem & Varmus, 1987; Kao & Chen, 2002; Su & Yee, 1992; Tiollais et al., 1985). HBV is a prototype of the *Hepadnaviridae* family and replicates almost exclusively in the liver. HBV contains a 3.2 kb partially double-stranded DNA virus with four major open reading frames (ORFs) encoding surface antigens (preS1, preS2, and S proteins), core antigens (preC and C proteins), polymerase (P proteins), and X proteins. The HBV genome contains important promoters and enhancers that regulate viral replication. HBV gene expression is controlled by preS, S, preC/C, and X promoters in the HBV genome, which are regulated by enhancers I and II (Antonucci & Rutter, 1989; Yu & Mertz, 2001; Yuh & Ting, 1990). These promoters and enhancers are regulated by a variety of factors. In previous studies, a variety of host transcription factors, including nuclear receptors (NRs), have been defined as regulators of HBV gene expression (Ganem & Varmus, 1987; Hu & Siddiqui, 1991; Zheng et al., 2004). The farnesoid X receptor (FXR) is a nuclear receptor that is mainly expressed in the kidney, small intestine, and liver (Fiorucci et al., 2007; Kuipers et al., 2004; Quasdorff & Protzer, 2010; Ramière et al., 2008).

FXRα encodes four isoforms of transcripts (FXRα1–4), resulting from alternative splicing and the use of two distinct promoters that initiate transcription. Notably, the expression of four FXR isoforms shows a tissue-specific pattern along the gut-liver axis, and FXR target gene expression is isoform-dependent. The two FXR genes were designated FXRα (NR1H4) and FXRβ (NR1H5). FXRβ is a pseudogene in humans, but its physiological function remains unclear. FXRα (also referred to as FXR) is highly expressed in the enterohepatic system, functions as a bile acid sensor and maintains cholesterol and bile acid homeostasis (Cariou & Staels, 2007; Fiorucci et al., 2007; Lee et al., 2006; Ramos Pittol et al., 2020). Several studies have demonstrated that FXR increases HBV gene expression (Bar-Yishay et al., 2011; Quasdorff & Protzer, 2010; Ramière et al., 2008).

The family of fibroblast growth factors (FGFs) has 22 members and plays important biological roles in differentiation, angiogenesis, cell growth, wound healing and repair, embryonic development, and metabolic regulation. FGFs are classified into canonical hormone-like FGFs (hFGFs) and intracellular FGFs (iFGFs). FGF11 is an iFGFs that functions intracellularly and is independent of the FGF receptor (Itoh & Ornitz, 2011; Nam et al., 2017). In contrast to other iFGFs, the function and molecular mechanisms of FGF11 activity have not been well studied. Recently, we reported that FGF11 regulates the expression of PPARγ by modifying the expression of multiple PPARγ regulators in adipogenesis (Lee et al., 2019).

This is the first report of FGF11 as a novel inhibitory cellular regulator of HBV gene expression. FGF11 decreases HBV promoter transactivation and mRNA and protein expression of HBV genes via FXRα inhibition. Importantly, these studies may contribute to the discovery of better regimens for the treatment of chronic HBV infections by including FGF11 to counter-act the bile acid-mediated FXR pathway.

## Materials and Methods

### Cell Culture

HepG2, Hep3B, and Chang liver cell lines (all from the American Type Culture Collection) were maintained in Dulbecco’s modified Eagle’s (DMEM) with 10% heat-in-activated fetal bovine serum (FBS) (GIBCO BRL) and 1% (v/v) penicillin–streptomycin (PS) (GIBCO BRL) at 37 °C in a humidified atmosphere containing 5% CO_2_.

### Plasmid Constructs and Reagents

Cp-luciferase HBV (1.3 ×) was provided by Y. Shaul (Weizmann Institute of Science). The 1.2 mer HBV (HBx+) replicon and HBV 3 × flag (1.2 mer HBV constructs including N-terminal 3 × flagged HBx) were provided by W. S. Ryu. pFlag-CMV2-human FGF11 (flag-h.FGF11) expression plasmid was provided by KIOST (Korea Institute of Ocean Science and Technology). The transfection reagents, jetPEI and jetOPTIMUS, were purchased from PolyPlus Transfection.

### Chemical Treatment

GW4064 was purchased from Sigma and prepared in dimethyl sulfoxide (DMSO) as a 10 mM stock solution. Z-guggulsterone, an antagonist of FXR (a nuclear receptor of bile acids), was purchased from Sigma and prepared in dimethyl sulfoxide as a 10 mM stock solution. For protein stability analysis, Hep3B cells were treated with the protein translation inhibitor cycloheximide (Sigma) or vehicle controls and incubated for 1 and 2 h before harvest. The concentration of cycloheximide used was 10 μg/ml. The control vehicle treatment (DMSO) was equivalent to the dose-range experiments for each of the tested chemicals. MG-132 (Calbiochem) was dissolved in DMSO and incubated for 1 and 2 h before harvest. MG-132 was applied at a concentration of 25 μM.

### Luciferase Assay

Cells were seeded in 24-well culture plates and transfected with the reporter vector and expression plasmid using jetPEI (PolyPlus). pFlag-CMV2 plasmid was added to achieve the same total amount of plasmid DNA transfection. After transfection, cells were rinsed with ice-cold PBS and lysed with 1 × cell culture lysis buffer (Promega). Luciferase activity was determined using an analytical luminometer, according to the manufacturer’s instructions. Luciferase activity was normalized to the transfection efficiency using the corresponding β-galactosidase activity. All assays were performed at least in triplicates.

### RNA Isolation, RT-PCR and Quantitative Real-Time-PCR

Total RNA was isolated from the cells using TRIzol reagent (Invitrogen) according to the manufacturer’s recommendations. cDNA was synthesized from 0.5 μg of total RNA using Moloney-murine-leukemia virus (M-MLV) reverse transcriptase (Enzynomics) and random hexamer at 37 °C for 1 h. A one-twentieth aliquot of cDNA was subjected to PCR amplification using gene-specific primers. The cDNA was amplified by PCR and the PCR products were examined by electrophoresis on a 1.5% agarose gel. RT-PCR bands were quantified relative to the GAPDH control band. qRT-PCR was performed using the TOPreal qPCR 2 × PreMIX with SYBR Green (Enzynomics). The comparative Ct method was used to calculate relative gene expression levels, with GAPDH as an endogenous control gene.

### SDS-PAGE and Western Blotting

Cells were prepared by washing with cold PBS before lysing with lysis buffer (150 mM NaCl, 1% Nonidet P-40 [NP-40], 1 mM EDTA, 50 mM Tris, pH 7.5, 10% glycerol, 20 mM NaF and 5 mM Na_3_VO_3_) containing protease inhibitor and 1 mM PMSF (Phenylmethylsulfonyl fluoride). The protein concentration was determined using Bradford reagent (Bio-Rad), and BSA was used as a standard. Equal amounts of proteins were loaded and separated by SDS-PAGE (10–15%), and the proteins were transferred to polyvinylidene fluoride (PVDF) membranes (Millipore). For western blotting, the membranes were incubated with anti-actin (A2066, Sigma), anti-FLAG (#2368s, Cell Signaling Technology), anti-FXR (#72105s, Cell Signaling Technology), anti-HA (1 867423, Roche) antibodies in TBST (Tris-buffered saline containing 1% Tween 20) supplemented with 3% non-fat dried skim milk overnight at 4 °C. After washing three times with TBST, the blotted membranes were incubated with a peroxidase-conjugated secondary antibody (Enzo) for 40 min at room temperature. The proteins were visualized using an enhanced chemiluminescence (ECL) development reagent (Amersham Pharmacia Biotech).

### RNA Interference and Transfection

For siRNA-mediated downregulation of FXR, gene-specific siRNA and negative control siRNA were purchased from Santa Cruz Biotechnology and Bioneer. Hep3B cells were transfected with jetPRIME (Polyplus-Transfection SA) according to the manufacturer’s instructions.

### Co-immunoprecipitation

The cells were lysed in radioimmunoprecipitation assay buffer. Cell lysates were mixed with an anti-HA antibody (1 867423, Roche) or anti-FLAG antibody (#2368s, Cell Signaling Technology) at 4 °C for 12 h with gentle agitation. Immune complexes were collected on protein G-Sepharose beads (Invitrogen) and incubated for 2 h in a cold room. After washing three times with RIPA buffer, the precipitates were boiled with an equal volume of 2 × Laemmli sample buffer, separated by SDS-PAGE, and subjected to western blot analysis.

### Hydrodynamic Injection (HI) in Mice

Eight-week-old C57Bl/6J mice were hydrodynamically injected with 10 μg of plasmid DNA (1.2 mer HBV [HBx+] replicon) and adenoviruses into the tail veins in a volume of PBS equivalent to 10% of mouse body weight. Adenoviruses encoding human FGF11 (Ad-FGF11) and control adenoviruses (Ad-GFP) were provided by KIOST (Korea Institute of Ocean Science and Technology). The liver tissue was taken from the mice receiving HI at 7 days post injection, which was then used for HBV DNA detection through quantitative real-time PCR.

### Statistical Analysis

The statistical software GraphPad Prism 5.03 (GraphPad Software, Inc.) was used for the analysis. All experiments are presented as mean ± standard deviation (SD) and were analyzed by one-way analysis of variance (ANOVA) followed by Tukey’s post hoc test. Statistical significance was set at *p* < 0.05.

## Results

### FGF11 Decreases HBV Gene Expression

**Fig. 1:**
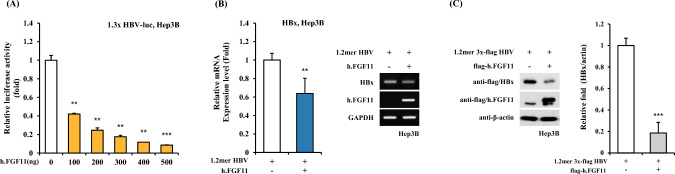
FGF11 inhibits HBV promoter activity and gene expression of HBx. **A** Hep3B cells were co-transfected with the 1.3 × HBV-luc construct and the FGF11 expression plasmids in a dose-dependent manner. After 24 h of transfection, cell lysates were obtained, and luciferase activity was measured. Data shown are means ± SD of three independent experiments performed in duplicate. **p < 0.01, ***p < 0.001 compared with control. **B** Hep3B cells were transfected with the 1.2 mer HBV, pFlag-CMV2-FGF11 of human. At 24 h post-transfection, the total cellular RNA was extracted from the cells, and the levels of HBx and GAPDH were analyzed by real-time PCR. Data shown are means ± SD of three independent experiments performed in duplicate. **p < 0.01 compared with control. **C** Hep3B cells were transfected with 1 μg each of 1.2 mer 3 × flag HBV, pFlag-CMV2-FGF11 with the indicated combination. Whole cell lysates were analyzed for the expression of the indicated proteins by western blot with anti-flag antibody. Right panels indicate a relative fold of western blot results. ***p < 0.001 compared with control

In a previous report, FGF11 was associated with the regulation of PPARγ and C/EBPα, which are known transcription factors that act on the HBV promoter. To investigate whether FGF11 regulates HBV gene expression, we applied transactivation assay using 1.3 × HBV-luc, which is a luciferase reporter construct including HBV promoter and enhancer, in the increasing expression of FGF11 in Hep3B cells. As shown in Fig. 1A, FGF11 gradually decreased the promoter transactivation of 1.3 × HBV-luc in an FGF11-expression dependent manner. Furthermore, consistent with the results shown in Fig. 1A, FGF11 also decreased mRNA expression (Fig. 1B) as observed via RT-PCR, and protein expression (Fig. 1C) observed using western blot assay with HBx, which is one of the four HBV-encoding proteins. These results indicated that FGF11 plays a critical role in HBV gene suppression by inhibiting HBV promoter transactivation.

### FXRα1 is Responsible for FGF11-Mediated Inhibition of HBV Gene Expression

**Fig. 2:**
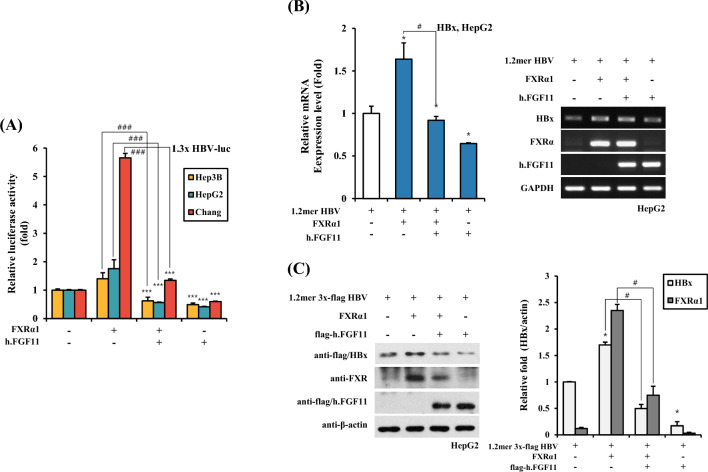
FGF11 suppresses FXRα-mediated gene activation of HBV promoter and HBx expression. **A** FXRα increases HBV promoter activation and FGF11 decreases HBV activation by FXRα. Hep3B, HepG2 and Chang cells were co-transfected with the 1.3 × HBV-luc construct, along with FXR and FGF11 expression plasmid as indicated combination. After 24 h of transfection, cell lysates were obtained, and luciferase activity was measured. Data shown are means ± SD of three independent experiments performed in duplicate. ***p < 0.001 compared with control. ^###^*p* < 0.001 compared with FXRα1 group. **B** HepG2 cells were co-transfected with the 1.2 mer HBV, pCMV-FXR, pFlag-CMV2-FGF11 with the indicated combination. At 24 h post-transfection, the total cellular RNA was extracted from the cells, and the levels of HBx, FXRα, FGF11 and GAPDH were analyzed by real-time PCR. Data shown are means ± SD of three independent experiments performed in duplicate. *p < 0.05 compared with control. ^#^*p* < 0.05 compared with FXRα1 group. **C** The HepG2 cells were co-transfected with the 1.2 mer 3 × flag HBV, pCMV-FXR, pFlag-CMV2-FGF11 of human with the indicated combination. Western blotting was performed on the cell extracts using anti-flag and anti-FXR serum. The equivalence of protein loading in the lanes was verified using anti-actin serum. Right panels indicate a relative fold of western blot results. *p < 0.05 compared with control. ^#^*p* < 0.05 compared with FXRα1 group

We aimed to identify the mechanism by which FGF11 inhibits HBV promoter transactivation. FXRα1 is known to increase HBV gene expression by acting positively on the HBV promoter. To analyze whether FXRα1 is associated with the inhibition of HBV transactivation by FGF11, we co-transfected the FXRα1 and FGF11 genes in combination with three different hepatocyte cell lines (Hep3B, HepG2, and Chang). FXRα1 overexpression increased promoter transactivation of 1.3 × HBV-luc in all three cell types, with the highest activity observed in Chang cells (Fig. 2A). Ectopic FGF11 expression largely decreased the FXRα1-mediated transactivation of the HBV promoter (Fig. 2A). We confirmed the FGF11 effect on FXRα-mediated HBV transactivation on HBV mRNA and protein expression. As shown in Fig. 2B, FGF11 decreased HBx mRNA expression induced by FXRα1. Consistent with this, HBx protein induction by FXRα1 also decreased in the presence of FGF11 overexpression (Fig. 2C). These results suggest that FGF11 inhibits HBV gene expression at the transcriptional level by suppressing the FXRα1-mediated transactivation of the HBV promoter.

### FXRα1 Ligand and FGF11 Show Counterbalance Function for HBV Gene Expression

**Fig. 3:**
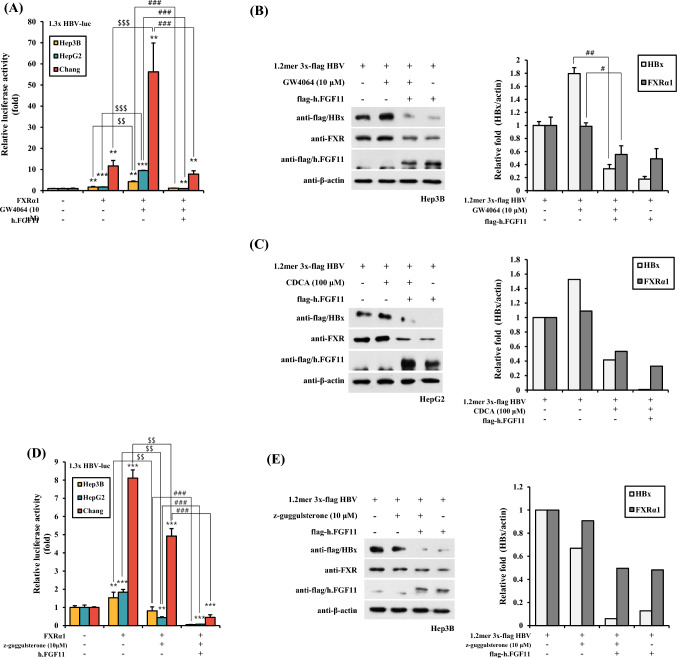
Agonists of FXRα largely increase HBV gene expression, but FGF11 inhibits FXRα agonist-derived HBV gene induction. **A** Hep3B, HepG2, and Chang cells were co-transfected with the 1.3 × HBV-luc construct, the FXR and FGF11 expression plasmid, and then maintained either under control conditions or in the presence GW4064 (10 μM) for 24 h. Data shown are means ± SD of three independent experiments performed in duplicate. **p < 0.01, ***p < 0.001 compared with control. ^###^*p* < 0.001 compared with GW4064 group. ^$$^*p* < 0.05 compared FXR versus FXR + GW4064. **B**, **C** The Hep3B cells were co-transfected with the 1.2 mer 3 × flag HBV construct and the FGF11 expression plasmid and maintained either under control conditions or in the presence of GW4064 10 μM (**B**) or CDCA 100 μM (**C**) for 24 h. Western blotting was performed on the cell extracts using anti-flag and anti-FXR serum. The equivalence of protein loading in the lanes was verified using anti-actin serum. Right panels indicate a relative fold of western blot results. ^#^*p* < 0.05, ^##^*p* < 0.01 compared with GW4064 group. **D** Hep3B, HepG2, and Chang cells were co-transfected with the 1.3 × HBV-luc construct, the FXR and FGF11 expression plasmid, and then maintained either under control conditions or in the presence of z-guggulsterone (10 μM) for 24 h. Data shown are means ± SD of three independent experiments performed in duplicate. **p < 0.01, ***p < 0.001 compared with control. ^###^*p* < 0.001 compared with z-guggulsterone group. ^$$^*p* < 0.05 compared FXR versus FXR + z-guggulsterone. **E** The Hep3B cells were co-transfected with the 1.2 mer 3 × flag HBV construct and the FGF11 expression plasmid and maintained either under control conditions or in the presence of z-guggulsterone (10 μM) for 24 h. Western blotting was performed on the cell extracts using anti-flag and anti-FXR serum. The equivalence of protein loading in the lanes was verified using anti-actin serum. Right panels indicate a relative fold of western blot results

FXRα1 plays a critical role in gene promoter transactivation as a transcription factor after binding specific ligand(s) to the ligand-binding domain (LBD) of FXRα1. We examined the effect of agonistic ligands on the FGF11-mediated transactivation inhibition of the HBV promoter. GW4064 is a synthetic agonist ligand that binds to the FXRα1 LBD. GW4064 treatment largely increased the FXRα1-derived transactivation of 1.3 × HBV-luc cells in all three hepatic cell lines (Fig. 3A).

To further confirm the FGF11 inhibitory effect on GW4064 agonist-derived HBV gene expression, we analyzed HBx protein expression in both FXRα1 expression and GW4064 treatment in combination with FGF11 expression. As shown in Fig. 3B, FGF11 overexpression decreased GW4064- and FXRα1-dependent HBx protein expression. In addition, we used the natural ligand CDCA for FXRα1 in the FGF11-mediated transcriptional inhibition of HBV. Consistent with the results shown in Fig. 3B, FGF11 clearly decreased HBx protein expression in CDCA-mediated HBx induction. These results indicate that FGF11 inhibits the agonist dependent FXRα1 transactivation of HBV gene expression.

In contrast to FXRα1 agonists, we examined the antagonist effect on FGF11-mediated transcriptional inhibition of HBV. For this purpose, Z-guggulsterone was used as an antagonist of FXRα1. While Z-guggulsterone treatment largely decreased FXRα1-mediated transactivation of the HBV promoter, FGF11 expression inhibited HBV promoter transactivation more dramatically (Fig. 3D). This transactivation inhibition effect was confirmed in protein expression experiments with western blot assay, which showed that both FGF11 expression and Z-guggulsterone treatment synergistically repressed HBx protein expression (Fig. 3E). These results indicate that FGF11 inhibits HBV gene expression by suppressing ligand-activated FXRα1 transactivation.

### Suppression of FXRα1 Expression and Its Transcriptional Function Inhibits FGF11 Effect on HBV Gene Expression

**Fig. 4:**
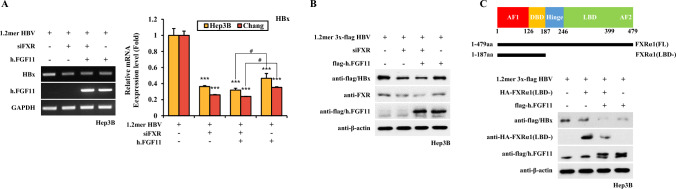
Reduced expression of FXRα selectively suppresses FGF11-mediated HBV inhibition. **A** FXRα siRNA counteracts FGF11-mediated HBx RNA transcription. Hep3B and Chang cells were transfected with pFlag-CMV2-FGF11 expression plasmids. At 24 h post-transfection, the total cellular RNA was extracted from the cells, and the levels of FGF11 and GAPDH were analyzed by RT-PCR (left) and quantitative real-time PCR (right). Data shown are means ± SD of three independent experiments performed in duplicate. ***p < 0.001 compared with control. ^#^*p* < 0.05 compared with FGF11 group. **B** FXRα siRNA decreases HBx expression. Hep3B cells were transfected with pFlag-CMV2-FGF11 expression plasmids. Western blotting was performed on the cell extracts using anti-flag serum. The equivalence of protein loading in the lanes was verified using anti-actin serum. **C** Dominant negative mutant (LBD deletion) expression of FXRα strengthens FGF11-mediated HBx reduction. Hep3B cells were transfected with pFlag-CMV2-FGF11 and HA-FXRα1(LBD-) expression plasmids. Western blotting was performed on the cell extracts using anti-flag and HA antibodies

To further confirm whether FXRα1 mediates FGF11-derived inhibition of HBV gene expression, we used siFXRα1 RNA. In the 1.2mer HBV replicon, siFXRα1 decreased HBx RNA expression by more than half (Fig. 4A). When the effect of FGF11 on HBx RNA expression was compared in the presence or absence of siFXRα1 expression, both FGF11 and siFXRα1 expression did not significantly change HBx RNA expression (Fig. 4A) With the confirmation of the decrease in levels of FXRαl by siFXRα1, HBx protein expression was also found to have largely decreased by siFXRα1. In the presence of siFXRαl, FGF11 did not significantly affect HBx expression (Fig. 4B). This result indicates that the inhibitory effect of FGF11 on HBx expression was dependent on FXRα1.

The DNA-binding domain (DBD) alone of nuclear receptors acts as a dominant negative mutant protein. As shown in Fig. 4C, we prepared the N-terminal (aa 1–187) domain, including DBD, an expression construct (FXRα1[LBD-]), and applied it to transient transfection in Hep3B cells in the presence or absence of FGF11 expression. FXRα1(LBD-) decreased FXRα1-mediated HBx protein induction, and FGF11 expression suppressed HBx regulation more than FXRα1(LBD-) alone (Fig. 4C). FXRα1(LBD-) binds to the FXR-responsive element of HBV and inhibits full-length FXRα1 from binding to the DNA element. Since not all endogenous FXRα1 proteins were affected by ectopic expression of the FXRα1(LBD-) protein as a dominant negative mutant, FGF11 inhibited the transactivation of the remaining FXRα1. These results indicate that FXRα1 is required for FGF11-mediated HBV gene expression.

### FGF11 Decreases Protein Stability of FXRα1

**Fig. 5:**
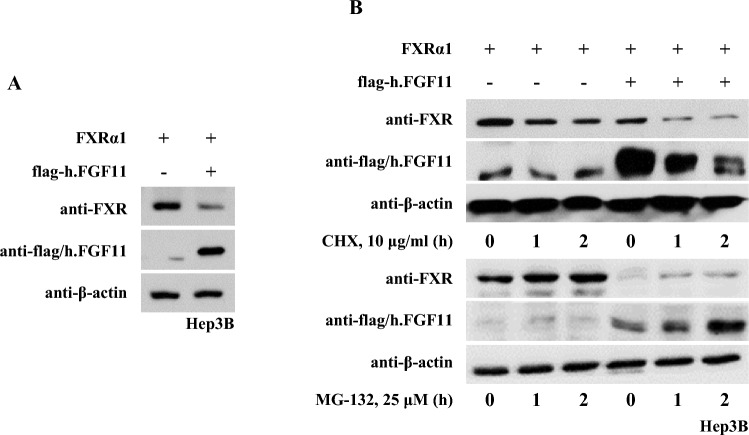
FGF11 decreases FXRα protein stability. **A** Ectopic FGF11 expression decreases cellular protein level of FXRα. Hep3B cells were transfected with pFlag-CMV2-FGF11 expression plasmids. After 24 h of transfection, western blotting was performed on the cell extracts using anti-FXRα antibody. **B** FGF11 expression decreases de novo protein synthesis of FXRα1. Hep3B cells expressing FXRα and flag-FGF11 were treated with 10 μg/ml CHX or 25 μM MG-132 for the indicated period. Whole cell lysates were analyzed for the expression of the FGF11 proteins by western blot

We examined the protein levels of full-length FXRα1 in the presence or absence of FGF11 protein expression. FGF11 expression in Hep3B cells significantly decreased the FXRα1 protein levels (Fig. 5A). Cycloheximide (CHX), an inhibitor of de novo protein synthesis, showed that protein degradation of FXRα1 was facilitated by FGF11 expression in a time-dependent manner (Fig. 5B). In addition, we used the proteasome inhibitor MG-132 for both FXRα1 transfection and CHX treatment in the presence or absence of FGF11 expression.

### FGF11 Protein Interacts with FXRα1 Protein

**Fig. 6:**
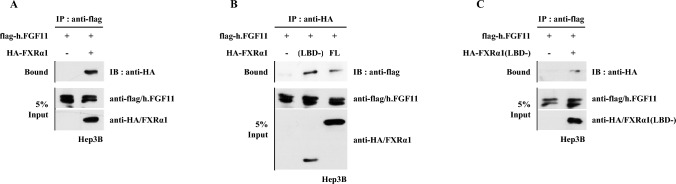
FGF11 interacts with FXRα through the N-terminal domain of FXRα. **A** FGF11 interacts with intact FXRα protein in the co-immunoprecipitation. **B**, **C** The N-terminal domain of FXRα is required for protein–protein interaction with FGF11. At 24 h post-transfection, cell lysates were collected for co-immunoprecipitation with beads conjugated with HA antibody (**B**) and flag antibody (**C**). The bound proteins were detected by western blot with flag antibody (**B**) and HA antibody (**C**)

The results of the transient transfection assay suggested that FGF11 represses transcription by directly binding to FXRα1. To determine whether FGF11 inhibition of FXRα1 is mediated by protein–protein interactions, we performed co-immunoprecipitation assays. Hep3B cells were transfected with FGF11 and/or FXRα1 expression vectors. Whole cell extracts were prepared and immunoprecipitated using either anti-FLAG or anti-HA antibodies. Immunoprecipitates were separated by SDS-PAGE and immunoblotted with anti-HA or anti-FLAG antibody. After co-immunoprecipitation with anti-FLAG to detect FGF11 and its associated proteins, a western blot assay was performed with anti-HA to detect FXRα1 as an associated protein with FGF11. As shown in Fig. 6A, the FXRα1 protein was detected among FGF11-interacted proteins. As shown in Fig. 4C, we used FXRα1(LBD-) as a dominant-negative mutant of endogenous FXRα1. In the co-immunoprecipitation assay, FGF11 interacted with both full-length FXRα1 and truncated FXRα1(LBD-) (Fig. 6B). We further confirmed that co-immunoprecipitation with FGF11 (using anti-FLAG antibody) contained the FXRα1(LBD-) protein as an FGF11-interacting protein (Fig. 6C). These results indicate that protein–protein interactions between FGF11 and FXRα1 lead to protein destabilization of FXRα1.

**Fig. 7:**
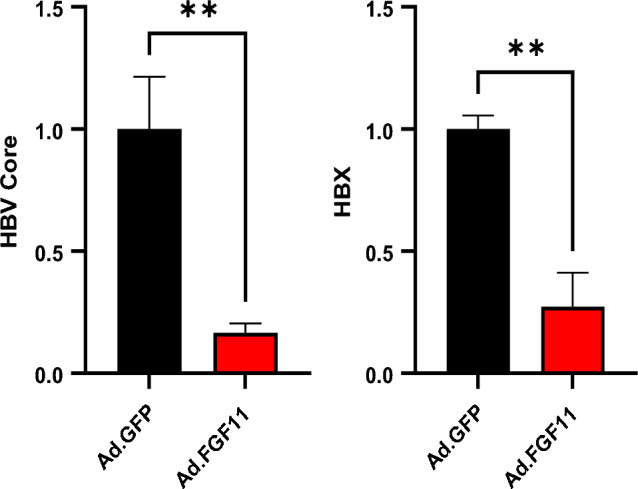
Inhibitory effect of FGF11 on HBV replication in in vivo mouse model. 10 μg of 1.2 mer HBV replicon and 1 × 10^11^ pfu of adenoviruses were injected into the tail veins of C57Bl/6J mice. Quantitative real-time PCR detection of HBV DNA in liver tissue at day 7 after HI. Data shown are means ± SD of independent experiments performed in duplicate. **p < 0.01 compared with control

In order to further confirm whether FGF11 inhibits HBV expression in vivo, we applied to HBV producing mouse by inserting 1.2mer HBV replicon in the presence or absence of adenovirus-FGF11 hydrodynamic injection (HI). After seven days HI of adenovirus-GFP (as a control) and adenovirus-FGF11, the liver tissues from both mouse were applied to quantitative real-time PCR detection of HBV DNA. As shown in Fig. 7, exogenous FGF11 expression from adenovirus-FGF11 dramatically inhibited both HBV core and HBx expression in the mouse livers. This result indicates that FGF11 decrease HBV gene expression in vitro and in vivo.

## Discussion

In this study, we investigated the antiviral effects of FGF11 on HBV expression. FGFs are required for organ development and regeneration but are also involved in metabolism. However, little is known about their role during viral infection. Recently study reported that FGF16 was identified as the most prominent novel inhibitor of vesicular stomatitis virus (VSV) and therefore of viral replication (van Asten et al., 2018). In addition, other members of the FGF family also inhibited viral replication. This previously unappreciated role of the FGF family may have implications for the development of new antivirals. Importantly, these studies may contribute to the discovery of better regimens for the treatment of chronic HBV infections by FGF11, which alters the host factor-mediated HBV replication pathway.

Substantial progress has been made in the treatment of chronic hepatitis B (CHB) over the last two decades. Currently, there are a number of approved drugs for the treatment of CHB, including two formulations of interferon (IFN)—conventional and pegylated IFN (PegIFN)—and six NAs: lamivudine, telbivudine, adefovir, entecavir, tenofovir disoproxil fumarate (TDF), and tenofovir alafenamide fumarate (TAF) (Eun et al., 2010; Lai & Yuen, 2013; Suk-Fong Lok, 2019; Yang et al., 2009). Several drugs that directly target the HBV replication cycle or enhance the human immune response are currently under development. A majority of individuals suffering with CHB will probably not benefit from current antiviral therapy. The quasispecies nature of populations of HBV in vivo implies that minor populations of drug resistant mutants are present even in treatment-naive patients, so institution of therapy is bound to force their selection and amplification. New drugs against HBV include agents that directly target the viral life cycle or indirectly modulate host factors for HBV replication/transcription (Morikawa et al., 2016; Suk-Fong Lok, 2019).

FGF11 is induced in endothelial cells by HIF-1α and stimulates capillary-like endothelial tube formation, which is associated with angiogenesis (Lee et al., 2017; Yang et al., 2015). Hypoxia is commonly observed in solid cancers and is particularly frequent in hepatocellular carcinomas because of its rapid growth. Compared with HIF-2α, the induction of HIF-1α expression was greater under low oxygen concentration, CoCl_2_, or DMOG treatments, indicating less involvement of HIF-2α (Befani et al., 2013; Keith et al., 2011; Nath & Szabo, 2012). It can be inferred that HIF-1α translocates into the nucleus and binds to hypoxic response elements in FGF11 induction, leading to a decreased HBV load and probably facilitating adaptation to HBV infection. The expression and activation of HIF-1α and FGF11 inevitably needs to be considered for evaluating prognosis and therapeutic options for HBV-derived hepatocellular carcinoma.

Bile acid CDCA and an artificial agonist GW4064 for FXRα increased HBV protein expression and promoter activation in HBV replicon-harboring cells (Fig. 3). Activation of FXRα by bile acids induces the expression of various proteins, including SHP, which represses the expression of cholesterol 7-hydroxylase, the rate-limiting enzyme in bile acid synthesis. The FXR/SHP pathway is well developed in hepatic, intestinal, and renal cells and participates in the regulation of fatty acid (including cholesterol) metabolism and glucose homeostasis (Calkin & Tontonoz, 2012; König et al., 2017; Reese et al., 2012). We tested whether this bile acid-mediated FXR pathway is important in bile acid-mediated HBV replication using z-guggulsterone, an antagonist of FXR. In the presence of z-guggulsterone, the bile acid-mediated increase in HBV promoter activation had reduced up to 50% of that observed with bile acids alone (Fig. 3). This finding suggests that the FXR pathway is important for bile acid-mediated HBV replication and FGF11-mediated inhibition of FXR activation suppresses HBV gene expression for viral replication.

Stability of FXRα protein is critical for their biological function and efficacy. Predicting the energetic effects of protein mutations can improve our fundamental understanding of structural biology, the molecular basis of diseases, and possible routes to address these diseases using biological drugs (Magliery, 2015; Steinbrecher et al., 2017). These effects of FGF11 on FXRα indicate a meaningful approach for anti-HBV drugs. FGF11 induction leads to FXRα protein instability by protein–protein interaction of FGF11 and FXRα, resulting in suppression of FXRα-mediated HBV pregenomic RNA transcription for viral multiplication. This study provides a more comprehensive description of the mechanism by which FGF11 inhibits HBV transcription and suggests that FGF11 is a promising treatment option for HBV.

## Data Availability

The datasets generated and analyzed during the current study are available from the corresponding author on reasonable request.
